# Aflatoxin tests in herbal products and its quantification: Latest updates

**DOI:** 10.3389/fnut.2022.956077

**Published:** 2022-09-08

**Authors:** Simon Vienoth Victor Jeyaraj, Mei Jun Loy, Khang Wen Goh, Yen Loong Lean, Siok Yee Chan, Long Chiau Ming

**Affiliations:** ^1^Faculty of Pharmacy, Quest International University, Ipoh, Malaysia; ^2^Faculty of Engineering, Universiti Teknologi Malaysia, Skudai, Malaysia; ^3^Faculty of Data Science and Information Technology, INTI International University, Nilai, Malaysia; ^4^School of Pharmaceutical Sciences, Universiti Sains Malaysia, Minden, Malaysia; ^5^PAP Rashidah Sa'adatul Bolkiah Institute of Health Sciences, Universiti Brunei Darussalam, Gadong, Brunei

**Keywords:** international health regulations, sustainable food production, contamination, food safety, patient safety

## Abstract

Aflatoxin is naturally occurring mycotoxins produced by fungi. The existence of aflatoxin in herbal medicines is a well-known issue. The detection of aflatoxin with good sensitivity and also that is reliable in complex matrices like herbs usually necessitates difficult processes and powerful detection instrument in preparation of sample. This study investigated the global occurrence of aflatoxin contamination herbal products. This article pivots on key breakthroughs in preparation of sample and its importance in analytical technology. Studies from published studies were screened to determine the general level of aflatoxin contamination. The countries involved were Malaysia, Indonesia, Kenya, Brazil, Nigeria, Thailand, South Africa, and Morocco. This review also includes recent studies on the development and application of screening assays such as lateral flow immunoassays, enzyme-linked immunosorbent assays, aptamer-based lateral flow assays, and cytometric bead arrays, as well as traditional chromatographic techniques for aflatoxin qualification or quantitation. The current study looks at aflatoxin contamination of key herbal drug raw material, which are frequently used in the production of numerous herbal pharmaceuticals. Contamination of aflatoxin might occur in herbal products if the ingredients such as medicinal herbs and plants that are used in manufacturing of herbal products are not dried thoroughly or stored inappropriately after preparation.

## Introduction

Aflatoxin are mycotoxins that naturally occurring which are produced by certain fungi like *Aspergillus Parasiticus* and *Aspergillus Flavus* (1, 2). Both of these fungi are quite commonly found in our environment (3). These fungi are also found in drought like stressful conditions because drought stress can increase the contamination of aflatoxins in certain grains like maize, sorghum and even groundnuts (4, 5). They can be found in soil, decaying vegetation, hay and grains that microbially decay, and invades all types of organic substances in conditions that has the presence of high moisture content and high temperature (6, 7). There are about 13 different types of aflatoxin produced naturally (8, 9). However, researchers have found that Aflatoxin B_1_ is the most toxic with carcinogenic, teratogenic and mutagenic properties (3, 10). Aflatoxin is confirmed as a Group-1 agent based on the evaluation done in IARC Monograph Volume 82 (2002) (11). Aflatoxin was classified as Group-1 carcinogens based on the fact that aflatoxin exhibits a significant increase in risks for hepatocellular carcinoma (HCC) among those have exposure to aflatoxin in statistics, as measured using aflatoxin-specific biomarkers in cohort studies in Taiwan and Shanghai, China (11). This significance was when there is no exposure to hepatitis B virus (HBV) (11). However, a higher than multiplicative interaction was noted between aflatoxin exposure and HBV infection when HBV infection was taken into account (11). There are more than 75 countries that regulates Aflatoxin (10). The most found limits for aflatoxin B1 and total aflatoxin in food are 5 and 20 μg/kg, respectively (10). European Union countries' limits for aflatoxin B1 and total aflatoxin in food are 2 and 4 μg/kg, respectively (10).

Traditional herbal medicines are gaining popularity in a fast phase throughout the world due to the increase in market presence of food supplements and its sales has grown year on year (10). Certain countries like Malaysia, Indonesia and Thailand has identified the herbal industry as a new source of economic growth contributor (12). Herbs have been classified as potential agricultural commodities and is expected greatly contribute to gross domestic product of a country and create job opportunities for its people (12). For example, herbal industry has been chosen as the first in Entry Point Project (EPP1) under Malaysia's Agriculture New Key Economic Area (12). The herbal medicine sector was valued at US$83 billion in 2019 and is expected to reach US$550 billion in 2030 (12, 13). The global market for herbs and herbal products has grown significantly in the last 10 years, and Malaysia has felt the effects (12). Malaysia, despite its biodiversity, is a net importer of herbal items (12). Between 2011 and 2017, the average value of imports climbed by around 4%, while the average value of exports increased by about 10%. In 2016, and 2017, the biggest import and export values were US $ 547 million and US $ 174.8 million, respectively (12). In recent years, however, toxicity associated with herbal medications has become more of a worry, due to their increasing use in developed countries (10). Herbal products are manufactured from various ingredients that are naturally found. These naturally found ingredients could include farm produces. Therefore, aflatoxin contamination at preharvest, during harvest and after harvest must be given significance. Harvests like maize, sorghum, teff, wheat, barley and finger millet have documented mycotoxin contamination in a study done in Ethiopia (14).

The safety of herbal products manufactured from mycotoxin contaminated harvests threatens the safety of consumers throughout the world. Guidelines, evaluations and directives related to herbal medicines have been publishes to ensure the safe use of these products (10). If medicinal herbs and plants used in herbal products are not thoroughly dried or stored improperly during preparation, aflatoxin contamination can occur (10). Aflatoxins growth or contamination can be controlled during the harvesting and storing by using Good Agricultural Practices (GAPs), during preharvest and harvest practices. One of the specific areas that require GAPs is during grain handling where high moisture grain should not be held in wagons or trucks for a period of more than 6 h and grains with high moisture content should be dried using forced air to maintain cool temperature (15). Aflatoxin growth in grains dried using column dryers that are operated at temperatures between 180–200°F for a short duration of 1–2 h are low compared to layer-in-bin drying (15). Batch-in-bin drying is a much better method compared to layer-bin-drying to prevent aflatoxin growth (15). Farms in developing countries may not have column dryers and this is where drying temperature and drying time using layer-in-bin drying and batch-in-bin-drying plays a crucial role (15). Slow drying with low heat over long periods could promote aflatoxin development (15). The uncertainties in the management of harvests in different farms is the reason a rapid, economical, and accurate method to determine aflatoxin is necessary. As a rule of thumb, aflatoxin growth is prevented when the packaging is tightly sealed and there is no moisture content to cause aflatoxin growth. Unless the monograph specifies otherwise, herbal drugs cannot contain more than 2 μg/kg of aflatoxin B1 according to the British Pharmacopeia. For herbal product registration, most drug registration guidance documents include quality control of the standardized extracts and finished products where test for aflatoxin is required (16).

### Contamination of aflatoxin in herbal products at various countries

Ali et al. (10) conducted a study in 2005 to evaluate a method of determining the national occurrence of aflatoxin in traditional herbal products found in the commercial market of Malaysia and Indonesia. A total of 23 commercial traditional herbal products were sampled and the presence of aflatoxin was analyzed (10). The contamination level of aflatoxin were relatively low; the mean of the Aflatoxin B1 concentration of the 23 commercial traditional herbal products sampled in the study was 0.26 μg/kg (10). However, the aflatoxin levels obtained and the daily consumption of this herbal products gives a mean probable daily intake of 0.022 μg/kg (20% of tolerable daily intake) when calculated in this study (10). This shows that consuming this contaminated herbal product may represent an alternative route of aflatoxin exposure because herbal products are usually consumed frequently and for a prolonged duration (10). The study also concluded that further study that covers a large number of herbal products is required to establish the contamination level of aflatoxin in herbal products (10). Aflatoxin poisoning cases has occurred twice in Malaysia. One of the incidents is very serious till innocent young children have lost their lives. Contamination of aflatoxin in herbal products does occur. The most common method used for determining aflatoxin in herbal product is using high-performance liquid chromatography that is coupled with mass spectrometry. The samples used for each analytical technique has to be properly sampled, extracted and cleaned up based on the property of the sample and the analytical method used to ensure accurate aflatoxin analysis results are obtained.

Zhang et al. (17) conducted a study on quality of herbal medicines in 2012. It was stated in this study that contamination by fungi and mycotoxins such as aflatoxin in herbal products was found in Malaysia (17). The drug registration guidance document that is published by the National Pharmaceutical Regulatory Agency of Malaysia who is equivalent to FDA in United States of America revealed that aflatoxin levels should be declared under quality control of the standardized extracts and finished products registration process (16). Currently, aflatoxin test is not mandatory for herbal products applying for registration in Malaysia. Ministry of Health, Malaysia only conducts continuous monitoring of foods that have notable risks to Aflatoxin contamination and to ensure that the aflatoxin contamination is within the permitted level and the foods are safe for consumption.

Keter et al. (18) conducted a study on risk of fungi such as Aflatoxin and Fumonisin that can be found in medicinal herbal products in the Kenyan Market. It was concluded in this study that 69% of the herbal products sampled has fungal limits that that are higher than the fungal acceptable limits stated in international Pharmacopeias; Aflatoxin levels ranged from 1 to 24 ppb, while Fumonisin levels ranged from 1 to >20 ppb (18). It was stated in this study that 42.9% of liquid samples from Eldoret had aflatoxin contamination and 100% of liquid samples from Mombasa were contaminated with aflatoxin (18). In addition, all 3 oil samples from Mombasa were contaminated with aflatoxin (18).

Bugno et al. (19) conducted a study on occurrence of toxigenic fungi in herbal drugs (*n* = 91) that are found in the Brazilian market. It was concluded in this study that 54.9% of the medicinal plants that were analyzed had higher levels than the fungal acceptable limit outlined in United States Pharmacopeia (USP) (19). The study found the presence of Aspergillus isolated that has the ability to produce mycotoxins such as aflatoxin (19).

Ezekwesili-Ofili et al. (20) conducted a study on the bioload and content of aflatoxin in herbal medicines found in certain states in Nigeria. It was found in this study that Aflatoxin B1 was the most commonly recorded mycotoxin in the samples that is above the acceptable levels (18.6%) outlined by the World Health Organization (20).

Tassaneeyakul et al. (21) conducted a study on contamination of aflatoxin in herbal medicinal products that can be found in Thailand. A total of 28 herbal medicinal products were sampled and their aflatoxin contamination levels were investigated in this study (21). In term of result, 18% of samples were found to be contaminated with detectable amount of aflatoxin (21).

Katerere et al. (22) conducted a survey on mycological, Fumonisin and Aflatoxin contamination in traditional herbal medicines that are sold in South Africa. The fungal and also mycotoxin contamination of herbal products sold in Tshwane and Cape Town was assessed (22). At least one of the three fungi Aspergillus, Fusarium, and Penicillium was detected in 15 of the 16 samples studied (94 percent) (22). None of the samples were detected with aflatoxin (22). However, Aspergillus can produce mycotoxins such as aflatoxin (19).

Mannani et al. (23) conducted an assessment of herbal green teas in the Moroccan market and its aflatoxin levels. A total of 129 samples were taken of herbal green teas available in the Moroccan market for this study (23). Seventy-six samples out of the 129 samples (59%) in this study were detected of contamination with aflatoxin (23). 38 samples surpassed the maximum values of 10 ng/g and 12 samples above the limit levels of 5 ng/g imposed by Moroccan health regulations for total aflatoxin and aflatoxin B1 levels, respectively (23). The studies based on countries are summarized in Table 1.

**Table 1:** Reported contamination of aflatoxin in herbal products at various countries.

**Country**	**Year reported**	**Tested item**	**Summaries**	**Percentage of aflatoxin contamination**	**References**
Malaysia, Indonesia	2005	23 commercial traditional herbal products	Aflatoxin was found in a number of samples from a total of 23 commercial traditional herbal medicines collected in Malaysia and Indonesia.	20%	(10)
Kenya	2017	100 herbal products	69% of the herbal products sampled has fungal limits that that are higher than the fungal acceptable limits stated in international Pharmacopeias.	69%	(18)
Brazil	2006	91 medicinal plants	The fungal acceptable level established in the United States Pharmacopeia was not met by 54.9 percent of the medicinal plants tested.	54.9%	(19)
Nigeria	2014	210 herbal medicines	The most common mycotoxin found in the samples was Aflatoxin B1, which was found to be over the World Health Organization's permitted levels.	18.6%	(20)
Thailand	2004	28 herbal medicinal products	Aflatoxin was found to be present in 18% of the samples tested.	18%	(21)
South Africa	2008	16 herbal medicinal products	At least one of Aspergillus, Fusarium, or Penicillium was found in 15 of the 16 samples examined.	94%	(22)
Morocco	2020	129 herbal green teas	76 samples out of the 129 samples in this study were detected of contamination with aflatoxin.	59%	(23)

### Determination of aflatoxin in herbal products

The first step in determination of aflatoxin in herbal products is to do sampling and obtain a sample from a herbal product. The following stage is to remove as much mycotoxin, such as aflatoxin, as possible from the herbal product matrix into a solvent. The following step is a cleanup step of the solvent prior to aflatoxin analysis. Zhang et al. (24) did a thorough assessment of current methodologies for analyzing mycotoxins in herbal medicines as described below. British Pharmacopeia and European Pharmacopeia also has a standard method outlined for the determination of aflatoxin in herbal drugs. The analytical techniques of aflatoxin found in literature reviews include liquid chromatography, gas chromatography, lateral flow immunoassay (LFIA), enzyme-linked immunosorbent assay (ELISA), cytometric bed array and aptamer-based lateral flow assay (24). The analysis of chromatographic techniques used in aflatoxin testing is presented in Table 2.

**Table 2:** Analysis of chromatographic techniques used in aflatoxin testing.

**No**.	**Type of analytical method**	**Characteristics of analytical method**	**References**
1.	Total liquid chromatography	- Traditional method used for aflatoxin analysis.	(24, 25)
		- Low cost and no complex equipment required compared to high performance liquid chromatography.	
		- The results are based on visual comparisons and the intensity of spot which does not allow for precise quantitation of aflatoxin content.	
2.	High performance liquid chromatography	- Most common and widely recommended method in international pharmacopeias. (British Pharmacopeia, European Pharmacopeia)	(24, 26–28)
		- Requires expensive complex high performance liquid chromatography machines.	
		- Provides precise and accurate results computed by complex software and tools.	
		- The results can be used for accurate quantitation of aflatoxins.	
3.	Gas chromatography	- Requires mass spectrometers.	(24)
		- Requires additional derivatization step compared to liquid chromatography.	
		- High sensitivity results.	
		- Time consuming technique compared to liquid chromatography.	

### Thin layer chromatographic technique

Thin layer chromatographic has been the traditional method used for aflatoxin analysis since aflatoxin were discovered (24). The rise in demand for data accuracy, separation and quantitation of aflatoxin analysis made people to convert to high performance liquid chromatographic compared to thin layer chromatographic (24). Honma et al. (29) conducted a review that showed that aflatoxin B_1_ in maize samples were analyzed using thin layer chromatographic technique in 1978 (24). The study also showed that the usage of thin layer chromatographic technique has decreased to 48% in 1989 and to 7% in 2002. United States Pharmacopeia does recommend thin layer chromatographic technique for detection of aflatoxin in plant materials (24). Thin layer chromatographic technique is only used these days when there is low cost involved for analysis and low demand of equipment (24).

### High performance liquid chromatographic technique

The most frequent and widely used method for determining aflatoxin in herbal medicine matrices is high-performance liquid chromatography (HPLC), which is recommended in many international pharmacopeias (24). This is the method recommended in British Pharmacopeia and European Pharmacopeia for determination of aflatoxin in herbal drugs. The high-performance liquid chromatographic technique used for aflatoxin testing is usually with a fluorescence detector (24). In reverse-phase chromatography, Aqueous solvent mixtures dramatically reduce the fluorescence of aflatoxin B1 and aflatoxin G1 in reverse-phase chromatography (24). British Pharmacopeia have proposed post-column derivatization protocols (30). The process of pre-column derivatization was discovered to be a difficult method that could not be completed by an on-line operation (24). Post-column derivatization is reported more in literatures because of the complicated procedure of pre-column derivatization (24). British Pharmacopeia has proposed three types of post-column derivatization methods (30). Firstly, post-column derivatization with pyridinium hydrobromide perbromide. A pulseless pump and heating system is typically used (24). Second, British Pharmacopeia proposes post-column derivatization with photochemical reactor and thirdly with electrochemically generated bromine (30). Both of this type of post-column derivatization are much easier to be carried out compared to post-column derivatization with pyridinium hydrobromide perbromide (24). The sensitivity is also much higher and a wider linearity range is obtained from these two types of post-column derivatization (24).

Sample matrices frequently interferes with the retention times of analytes because the chemical constitution of herbal products can be complex (24). Further confirmation using more accurate detectors, such as mass spectrometry, can be used to counteract this interference (24). Mass spectrometry is typically utilized, however comparing chromatograms generated from high performance liquid chromatography with and without derivatization for the fluorescence intensities of aflatoxin B1 and aflatoxin G1 is another option (24). A study also discovered that altering the polarity of the mobile phase can solve the problem of interference (24). This way is just an alternative method for laboratories that do not possess mass spectrometers (24).

### Gas chromatographic technique

Gas chromatography is often utilized for determination of aflatoxin as an alternative approach, when mass spectrometers are utilized as detectors (24). The simultaneous qualitative and quantitative assessments of single or many analytes are possible with gas chromatography combined with mass spectrometry (GC-MS) (24). However, gas chromatographic technique requires an additional derivatization step that is acylation, is commonly required following sample clean-up step (24). The additional derivatization is necessary because some aflatoxin are not sufficiently volatile at the column temperature (24). High sensitivity was achieved using GC-MS method but no significant advances were reported using this method in aflatoxin analysis of herbal products (24). The reason behind this is probably the wide usage of liquid chromatography couples with mass spectrometry that does not require an additional derivatization step like gas chromatographic technique (24).

### Rapid screening techniques

Analysis of rapid screening techniques used in aflatoxin testing is summarized in Table 3.

**Table 3:** Analysis of rapid screening techniques used in aflatoxin testing.

**No**.	**Type**	**Characteristics of analytical method**	**References**
1.	Enzyme-Linked Immunosorbent Assay (ELISA)	- Most commonly used antibody-based assay currently.	(24)
		- High specificity	
		- Rapid detection	
		- Simple design	
		- Cheap	
2.	Lateral Flow Immunoassay (LFIA)	- Rapid detection	(24, 31, 32)
		- Simple operational method	
3.	Aptamer-based lateral flow assay	- Greater resistance toward harsh environments compared to lateral flow immunoassay	(24, 33)
		- Higher sensitivity	
		- Higher specificity	
		- Preferable for use in complicated matrices	
		- Rapid detection	
4.	Cytometric Bead Array	- New technology	(24, 34)
		- Early stage	
		- Good sensitivity	
		- Rapid detection	
		- Real-time analysis for trace small molecules (aflatoxins)	
		- Suitable for on-site high-throughput detection	
		- High cost	
		- Complex instruments required	

#### Enzyme-linked immunosorbent assay

Enzyme-Linked Immunosorbent Assay was first used officially in the early 1970s (24). The Enzyme-Linked Immunosorbent Assay is a detection assay that uses enzymes to catalyze the immunological reaction between an antigen and an antibody (24). Enzyme-Linked Immunosorbent Assay is the widely used antibody-based assay at the moment (24). The advantages of enzyme-linked immunosorbent assay include high specificity, rapid detection, simple design, and relatively cheap (24). There are some commercial enzyme-linked immunosorbent assay test kits available for aflatoxin (24). However, the majority of test kits are meant for testing of aflatoxin in food and not in herbal products (24). According to certain studies, the complex co-extract of herbal samples, especially analogs of analytes, may produce non-specific antibody reactions, resulting in an overestimation of contaminants at extremely low concentrations (24). Prior to the ELISA test, several research have suggested purifying the sample extract with a multipurpose column or an IAC (24). The use of ELISA as a qualitative preliminary screen has recently been proposed, with samples that test positive by ELISA being verified and exact contamination levels being quantified using LC-MS/MS or HPLC (24).

#### Lateral flow immunoassay

Lateral flow immunoassay is a technique that utilizes immunochromatographic test strips (24). This technique was initially developed in 1980. The advantages of this technique include rapid detection and simple way of use (24). This immunochromatographic strips mechanism of function depends on the travel of a particulate conjugate tag antibody to a specific antigen or vice versa on a porous membrane (24). Both the antigen and antibody need direct contact for detection (24). Lateral flow immunoassay has been found to be used to rapidly detect presence of aflatoxin in a variety of food commodities in a review conducted in 2013 (24). A colloidal gold test strip designed based on lateral flow immunoassay technique was used for the detection of aflatoxin B_1_ in lotus seeds in a study in 2015 (24). Aflatoxin contamination in Lotus seeds is extremely common (24). This approach recommended a simple sample pre-treatment phase that entailed extracting 5 g of each sample with 10 mL methanol-water (80:20, v/v) and diluting four times with PBS (pH 7.4) (24). The test strip's LOD was confirmed to be 2.5 ng/mL. There were no false positive or negative results after LC-MS/MS confirmation (24).

#### Aptamer–based lateral flow assay

The aptamer-based lateral flow assay is a rapid screening technique that is quite similar to lateral flow immunoassay (24). The most distinguishable difference is in this technique is that aptamers are used instead of antibodies (24). Nucleic acid-based aptamers are more resistant to harsh chemicals, high ionizing environments, pH, and organic solvents (24). Therefore, aptamers have higher sensitivity and specificity (24). Hence, Aptamers are preferred for usage in complex sample matrices (24). Aptamer-based lateral flow assays are created for the quick detection of aflatoxin B_1_ in corn samples (24).

#### Cytometric bead array

Cytometric bead array is a type of suspension array made up of optically encoded microbeads (24). This is a new technology and it is rapidly expanding for detection applications (24). The advantages of cytometric bead array include good sensitivity results, real-time analysis, and rapid detection for trace small molecules like aflatoxin (24). In addition, cytometric bead array is particularly preferable for on-site high-throughput detection (24). The application of cytometric bead array for aflatoxin analysis is still in its early stage due to its expensive set up and the very complex need of instruments (24).

## Conclusion

Aflatoxin contamination in herbal products is a serious matter and health authorities should make in mandatory for testing of aflatoxin in herbal products. Literature reviews in this study has enlightened that contamination of aflatoxin in herbal products has occurred in countries outside Malaysia such as Kenya, Brazil, Nigeria, Thailand, South Africa and Morocco. The importance of aflatoxin analysis in herbal products can be seen as international pharmacopeias such as British Pharmacopeia, European Pharmacopeia and United States Pharmacopeia have outlined methods of de termination of aflatoxin in herbal drugs. International Agency for Research on Cancer (IARC) has concluded that aflatoxin cause hepatocellular carcinoma. This is another reason why aflatoxin contamination in herbal products must be given significance. Herbal products are taken for long duration by humans and if the herbal products are contaminated with aflatoxin, consumers would have prolonged exposure to aflatoxin. The multiple cases of aflatoxin contamination in various countries reviewed in this article shows that significance should be given to contamination of aflatoxin in herbal products. A large-scale study on herbal products available should be carried out to address the lack of information on aflatoxin levels of herbal products found everywhere. The most suitable test method found in this review for determining aflatoxin levels in herbal products is high-performance liquid chromatography coupled with mass spectrometry. This method is not suitable to be used by everyone in the herbal industry especially farmers who produce the natural ingredients necessary for herbal products and domestic traditional medicine manufacturers. The rapid screening techniques reviewed in this article are part of techniques developed for mycotoxins but specific targeted rapid screening techniques for aflatoxin in complex herbal product samples are still not available commercially for the easy detection of aflatoxin by every party involved in the herbal product market. The development of tests to determine and quantify aflatoxin in herbal products should be much enhanced compared to the current rate of development reviewed in this article to address the increasing incidence of aflatoxin contamination in herbal products.

## Author contributions

SYC, LCM, and YLL conceived the concept. SVVJ and MJL wrote the initial draft and revised the manuscript. KWG, YLL, SYC, and LCM critically revised the manuscript and finalized the manuscript. MJL, SVVJ, and LCM significantly contributed to review the manuscript in reply to reviewers. All authors read and approved the manuscript.

## Conflict of interest

The authors declare that the research was conducted in the absence of any commercial or financial relationships that could be construed as a potential conflict of interest.

## Publisher's note

All claims expressed in this article are solely those of the authors and do not necessarily represent those of their affiliated organizations, or those of the publisher, the editors and the reviewers. Any product that may be evaluated in this article, or claim that may be made by its manufacturer, is not guaranteed or endorsed by the publisher.
